# Instrumented Walkway Gait Analysis Predicts Fallers in Neurological Disorders: Identifying Digital Biomarkers for Balance Monitoring

**DOI:** 10.3390/s26175644

**Published:** 2026-09-05

**Authors:** Victor S. You, Leland R. Barnard, Hugo Botha, Lauren M. Jackson, James H. Bower, Bryan T. Klassen, Benjamin D. Elder, Jonathan Graff-Radford, Charles L. Howe, Farwa Ali

**Affiliations:** 1 Department of Neurology, Mayo Clinic, Rochester, MN 55901, USA; you.sungmin@mayo.edu (V.S.Y.); barnard.leland@mayo.edu (L.R.B.); botha.hugo@mayo.edu (H.B.); jackson.lauren@mayo.edu (L.M.J.); bower.james@mayo.edu (J.H.B.); klassen.bryan@mayo.edu (B.T.K.); graff-radford.jonathan@mayo.edu (J.G.-R.); howe@mayo.edu (C.L.H.); 2 Department of Neurosurgery, Mayo Clinic, Rochester, MN 55901, USA; elder.benjamin@mayo.edu

**Keywords:** balance impairment, gait analysis, neurological disorder, machine learning, fall risk, rehabilitation

## Abstract

Assessing balance is crucial in neurological rehabilitation, yet while wearable sensors enable real-world monitoring, identifying reliable digital biomarkers remains challenging. This study utilized a high-fidelity instrumented walkway to determine which gait parameters best predict balance impairment, providing robust targets for future wearable applications. We analyzed 49 steady-state gait metrics from 140 individuals with diverse neurological conditions. Using statistical analysis and machine learning, we evaluated these parameters against objective force plate sway scores and clinical fall-history labels. Group analysis identified 16 parameters significantly distinguishing fallers from non-fallers, and a neural network classified fallers with an area under the curve of 0.75. Across all analytical approaches, overall gait variability, e.g., Stride Width S.D. and the Gait Variability Index, emerged as a universal predictor of balance impairment and fall risk. Furthermore, while traditional linear models emphasized spatial postural control, machine learning classification uniquely identified inter-limb asymmetry as a premier driver of fall prediction. These findings indicate that instrumented gait analysis effectively identifies digital biomarkers for balance deficits. Isolating these specific metrics provides a clear blueprint for meaningful metrics required for continuous objective monitoring and future development of personalized, adaptive rehabilitation strategies.

## 1. Introduction

The maintenance of balance is a cornerstone of human mobility and independence. Among aging and neurologically impaired populations, deficits in gait and, more so, balance control are a direct precursor to falls, which represent a major cause of morbidity, mortality, and loss of independence [1,2]. Gait refers to the biomechanics of walking measured using spatiotemporal parameters, such as velocity, stride length, etc., while balance refers to static and dynamic postural stability. Both gait and balance can be impaired in neurological diseases [3]. However, balance function is more strongly predictive of adverse outcomes such as falls compared to gait parameters like velocity on its own [4]. Moreover, dynamic balance measurements can detect abnormalities earlier in the course of neurological disease [5]. Thus, there is a strong clinical and research imperative for accurate, accessible, and meaningful balance assessment [6].

A variety of clinical gait and balance tests are available to assess individuals with neurological disease, such as the Gait Assessment and Intervention Tool, the timed 25-foot walk test, the Berg Balance Scale, the Balance Evaluation Systems Test (BESTest), and the Mini-BESTest [4,6,7,8]. Such clinical tests do not detect early or subtle changes in balance [5], do not quantify highly granular features of gait and balance [9], and often produce a qualitative or composite timed score to capture global functional status [10]. On the other hand, instrumented evaluations using devices and digital technology are more sensitive than clinical scales for evaluating subtle and early gait and balance deficits in neurological disease [11,12,13,14,15]. The gold standard methods for assessing balance are force plates, which assess center of pressure (COP), and marker-based three-dimensional motion analysis, which assesses center of mass (COM) dynamics and/or COP trajectory where integrated force plates are available, which provides highly precise and objective measurements of postural sway [16]. An example is the modified clinical test of sensory integration in balance (mCTSIB), which evaluates COP trajectory with eyes open and eyes closed using a force plate [17,18,19]. However, such assessments are typically conducted under static, laboratory-controlled conditions and may not represent balance function during walking, and access to such technologies may also be limited. Therefore, a few limitations of current assessments exist; clinical tests may lack sensitivity for early subtle deficits, whereas laboratory-based static stability assessments have limited generalizability to day-to-day walking, where most falls occur [20].

Recently, several studies have investigated predicting balance test performance using wearable devices, which are portable and easy to administer in real-life settings [21,22,23,24]. While earlier studies were often limited by small sample size and lack of validation [25], recent large-scale consortia efforts, such as MOBILISE-D [26], and relevant studies [27,28] have significantly advanced the in-lab validation of wearable sensors. Despite this progress, there is no standardized method for clinical sensor application; placing several wearable devices on patients can be time-consuming at the point of care, and data quality remains dependent on precise sensor placement. To overcome these challenges and enable reliable real-world monitoring, foundational research is needed to first identify the precise, highly sensitive “digital biomarkers” of balance impairment that next-generation wearables should target. A digital biomarker is a quantifiable, objective physiological or behavioral measure captured by digital sensing technologies, extending the traditional biomarker concept of an objective measured indicator of biological or pathogenic processes [29,30,31]. In mobility science, digital biomarkers are commonly organized into domains such as spatiotemporal gait parameters, gait dynamics/complexity, postural sway, and daily-life mobility metrics.

Recent studies employing machine learning for fall risk classification further underscore this need, demonstrating that when relying on standardized, clinically interpretable spatiotemporal gait features, models consistently yield moderate discrimination [32,33,34]. Higher classification performance is typically achieved only through opaque signal transformations or resource-intensive, raw-signal deep learning on multi-sensor, long-duration protocols.

An alternative approach to capturing dynamic balance control involves the use of instrumented walkways. Instrumented walkways capture subtle differences reliably and objectively [35,36,37]. Even though they require dedicated space, they are considered the gold standard against which other devices are validated, offering an unobstructed method to assess walking without the need to apply sensors, which currently face significant validation and standardization challenges [37,38]. Moreover, instrumented walkways significantly reduce the immediate assessment burden at the point of care, offering semi-automated data processing that significantly reduces clinician time [39,40,41]. Instrumented walkways serve as an ideal discovery platform even though instrumented walkways and wearable sensors serve distinct, complementary roles rather than being fully interchangeable [39].

In this context, instrumented walkways serve as an ideal discovery platform [39]. The rich dataset collected from a single, routine walking task presents a compelling opportunity: to investigate whether the detailed signatures of gait can be used to infer an individual’s underlying balance control. By extracting meaningful digital biomarkers from a high-fidelity reference system, we can create a blueprint for the precise parameters that wearable sensing technologies must capture to support individualized and adaptive rehabilitation.

In this article, we aimed to identify digital biomarkers for continuous balance monitoring by extracting gait parameters from instrumented walkway data that models normal daily walking. We investigated whether spatiotemporal parameters from a walkway could accurately predict objective balance performance from a force plate, as well as clinical fall-history. Ultimately, we performed statistical analysis and developed machine learning models to prove that these accessible gait parameters successfully detect underlying balance impairment and fall risk.

## 2. Materials and Methods

In this study, single-time-point data were obtained through a retrospective review of clinical records for patients who underwent clinical gait and balance assessments at the Mayo Clinic Neurology Gait Disorders Laboratory between February 2025 and July 2025. Initially, data from 187 individuals were collected during this period. Patients with clear neurological conditions, such as normal pressure hydrocephalus (NPH), cerebellar ataxia (CA), multiple sclerosis (MS), and Parkinson’s disease, were included in this study to isolate the effects of the primary neurological conditions, while participants with unclear or unknown diagnoses were excluded from the analysis. We also collected self-reported questionnaires about individuals’ perception of their walking and balance ability. The final cohort for analysis consisted of 140 participants with completed questionnaires including their falling history. As a preliminary cross-sectional observational study, power analysis was not conducted in the design. All study procedures and protocols were reviewed and approved by the Mayo Clinic Institutional Review Board (IRB) (Protocol No. 24-009292, approval date: 21 January 2024). Informed consent was obtained from all participants prior to their inclusion in the study.

The ProtoKinetic Zeno 20-foot walkway was used to collect gait data, and ProtoKinetics Movement Analysis Software (PKMAS, version 6.00c3e, ProtoKinetics LLC, Havertown, PA, USA) was used to verify single-foot fall-level data and extract gait features. All data used for analysis were collected during a single-day visit, meaning both the walkway gait assessments and the force plate balance tests were performed on the same day. During the assessment, participants were supervised by a physician and a technician at close range to ensure safety. Participants were instructed to walk back and forth at their normal day-to-day speed. To capture true steady-state gait and avoid the impact of acceleration and deceleration, participants made their turns off the pressure-sensitive walkway. Participants completed 5 to 7 continuous bidirectional passes across the mat, resulting in an actual evaluated walking distance of 100 to 140 feet. On average, participants completed 5.94 ± 1.63 passes, which yielded an average of 39.4 ± 11.5 steps per participant. This specific step volume was carefully chosen because it exceeds the minimum threshold recommended in the literature for calculating reliable steady-state gait parameters and variability, while effectively preventing patient fatigue [42,43,44].

If a participant used a cane or walker consistently in day-to-day walking, the gait test was performed with the patient’s gait aid to capture their daily walk features. Participants who reported the use of a wheelchair were able to complete the gait test with the help of an alternate gait aid such as a cane or walker. The PKMAS reliably processes continuous bidirectional walking and automatically labels contacts into footfalls or assistive device contacts by performing cluster analysis on the size, space, and timing of sensors activated in the mat. Furthermore, adding to the automated internal process, manual quality checks and reviews were performed by the examining physician to ensure high data quality and the accurate labeling of the right and left feet immediately post-acquisition.

For the balance assessment, the patients performed the modified Clinical Test of Sensory Integration and Balance (mCTSIB) [16] on the Bertec Functional 1000 Hz force plates. Participants stood on the force plate, keeping their feet shoulder-width apart, with eyes open for a total of three 10 s long trials for measurement of static sway. Participants, including those who typically require walking assistance, performed the force plate assessment without their gait aids to ensure valid center of pressure readings. To ensure participant safety during the test, clinical staff provided close stand-by guarding without making physical contact unless a loss of balance occurred.

Bertec Balance Advantage software (version 2.5.0, Bertec, Columbus, OH, USA) was used to collect and process the force plate data, computing the mean velocity of the Center of Gravity (COG) and the Sway Area of the COP as metrics for balance impairment. Within the Bertec Balance Advantage software, the continuous COG position is estimated from the COP time-series data using the following relationship based on the standard Inverted Pendulum Model: $COG=COP+\left(\frac{h}{g}\right)*{COP}_{acc}$, where ${COP}_{acc}$ represents the acceleration of the COP, g is the acceleration due to gravity, and h is the vertical height of the COG above the balance plate. The maximum value of these parameters measured was used for regression analysis. For the ground truth labeling of their balance impairment from the clinical perspective, patients were classified into two groups, non-faller and faller, based on their falling histories from self-reported questionnaires. Specifically, this classification was determined by assessing the number of unexplained or unprovoked falls in the past 12 months with the question: “How many unexplained or unprovoked falls have you had in the past year?” The terms unexplained and unprovoked were defined in the questionnaire, and a fall was defined as resulting from loss of balance or instability as opposed to a mechanical trip or slip. The questionnaire captured fall frequency in five categories: “zero”, “1 to 5”, “5 to 10”, “10 to 30”, and “more than 30”. Participants selecting “zero” were designated as non-fallers, while those selecting any of the subsequent categories were designated as fallers.

The PKMAS outputs 679 variables per gait trial. Based on prior clinical knowledge, we excluded technical/intermediate and redundant/highly correlated variables before the analysis and were left with the 49 features spanning the known gait domains, shown in Table 1. These not only include typical clinically meaningful spatiotemporal gait measures but also pressure- and foot-angle-derived ones that are of particular interest for balance, including those related to the center of pressure (COP), cyclogram intersection points (CISP), and angles between the feet and the mat, as well as the feet and the direction of progression (DOP).

We first performed regression analysis to directly investigate the relationship between balance impairment indicators, i.e., COG mean velocity and COP Sway Area, and gait parameters. We adjusted both gait features and balance impairment indicators by independently regressing out the effects of clinical covariates, such as sex, age, height, weight, and body mass index. Then, we used an external cohort from the Mayo Clinic Study of Aging [42], which is a population-based cohort (n = 5635) with an age distribution of 70.8 ± 13.0 years (range: 18–124), with gait data collected up to 2025, to normalize the 49 gait parameters via z-score normalization [43]. Based on the normalized gait parameters, we trained and evaluated the two regression models independently for each balance metric using the least absolute shrinkage and selection operator (LASSO) model [44] via leave-one-out cross-validation.

We performed a statistical analysis of gait parameters in relation to balance impairment labels from their falling history. We performed group analysis between “non-faller” and “faller” classes by performing a Welch’s *t*-test for each gait parameter. All statistical analysis was performed using IBM SPSS Statistics (Version 28, IBM Corp., Armonk, NY, USA). Also, we preliminarily developed classification models to investigate the predictability of balance impairment based on gait parameters for clinical screening. We used logistic regression with Least Absolute Shrinkage and Selection Operator (LASSO), random forest [45], support vector machine [46] with radial basis function kernel, and neural network [47] as classifiers. Using the aforementioned z-score normalized parameters, classification models (excluding the neural network) were trained and evaluated via leave-one-out cross-validation utilizing weighted loss inversely proportional to class frequencies. The neural network consisted of two hidden layers (256, 64 nodes) and applied the Adam optimizer [48] with a learning rate of 0.005 and a batch size of 32.

Lastly, we investigated gait parameters that showed importance in each experiment, i.e., direct regression of balance impairment indicators, group analysis, and predictive binary modeling of balance impairment, and their overlaps. From the regression task, we measured the regression coefficient and collected gait parameters with top coefficients. Similarly, we used the absolute value of the coefficient as feature importance for linear classification models. We computed the mean and standard deviation of the accumulation of the impurity decrease within each tree for the random forest [49]. We utilized permutation importance [49] to estimate feature importances for the non-linear models, such as SVM and neural network.

## 3. Results

### 3.1. Participant Gait Characteristics

The demographic and clinical characteristics of the 140 participants are summarized in Table 2. The cohort had a mean age of 67.0 ± 15.4 [years], with 48 females (34.3%) and 92 males (65.7%). The mean weight was 85.2 ± 19.8 [kg] and the mean height was 1.72 ± 0.10 [m], with a mean BMI of 28.7 ± 5.7. Our cohort is a clinical sample of neurological patients presenting to our neurological gait disorders laboratory. The strength of this cohort is in the range of neurological disorders represented, the assessment of walking as the patient does in day-to-day life on the instrumented walkway, improved ecological validity, and the generalizability of our findings across the assessed neurological disorders as opposed to just one disease category.

Participants presented with a range of neurological conditions; the most prevalent diagnoses were normal pressure hydrocephalus (n: 85, 60.7%), followed by cerebellar ataxia (n: 22, 15.7%), multiple sclerosis (n: 22, 15.7%), and Parkinson’s disease (n: 11, 7.9%). Based on the questionnaires, 69 subjects reported the use of gait aids in daily life. There were no statistically significant differences in age (*p* = 0.69), sex (*p* = 0.11), or general body metrics (weight, height, BMI) between the non-faller and faller groups. Furthermore, the distribution of primary neurological diagnoses did not significantly differ between the cohorts (*p* = 0.28). However, the faller group naturally showed significantly higher reliance on gait aids (*p* = 0.01) and reported significantly lower self-assessed gait confidence (*p* < 0.001) compared to non-fallers.

### 3.2. Regression Analysis of Force Plate Balance Indicators

**Figure 1:**
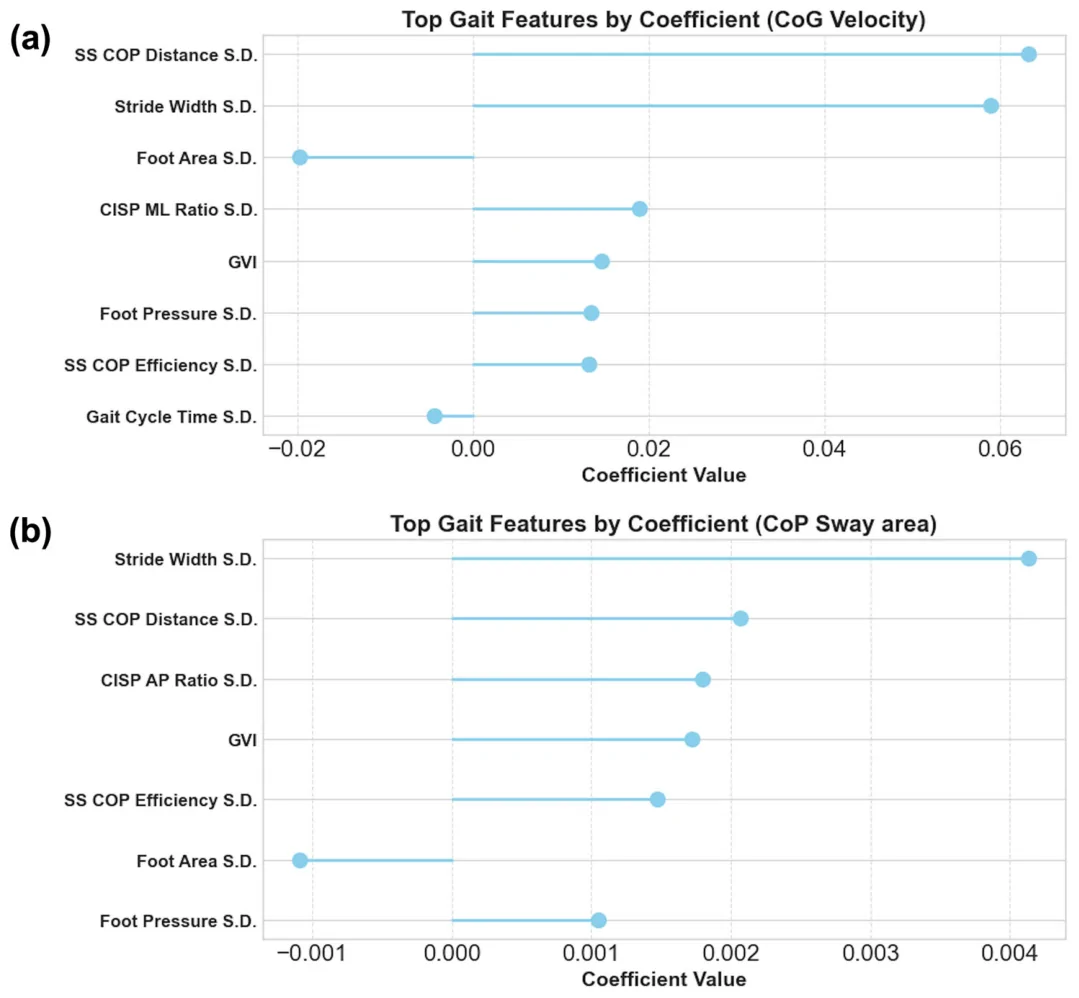
Top gait features from the direct regression of balance indicators (**a**) COG Velocity and (**b**) COP Sway Area. Gait features mostly related to gait variability, dynamic posture control, and based support, showed strong relationships with balance impairment metrics.

The LASSO regression models were trained to predict objective balance indicators from the force plate (COG mean velocity and COP Sway Area) using the selected 49 gait parameters after z-score normalization. Prediction of COG mean velocity: The model predicted COG mean velocity with a mean squared error (MSE) of 0.11 and an R^2^ of 0.37. The LASSO model selected 8 gait parameters as key predictors, including S.D. of SS COP Distance, Stride Width, SS COP Distance, Foot Area, and CISP ML Ratio. The top predictors from the LASSO model and corresponding coefficients are illustrated in Figure 1a. Prediction of COP Sway Area: The model for COP Sway Area achieved an MSE of 0.001 and an R^2^ of 0.24. Similarly, the model identified 7 gait parameters as key predictors, including S.D. of Stride Width, SS COP Distance, CISP AP Ratio, and SS Time. Likewise, the most influential predictors with corresponding coefficients are illustrated in Figure 1b.

### 3.3. Group Differences in Gait Parameters Between Non-Faller and Faller Groups

We compared the gait parameters between the “non-faller” (n = 61) and “faller” (n = 79) groups. Among the 49 target gait parameters, 16 were found to be significantly different between the two groups from Welch’s *t*-tests (*p* < 0.05).

First, gait parameters related to gait variability consistently showed that the fall-history group exhibited higher fluctuations across several parameters in standard stability measurements. Among them, the S.D. of CISP AP ratio showed the most pronounced difference, with a medium-to-large effect size (d=0.68, 95% CI [0.33,1.02]) and a mean difference of 1.25 units. Similarly, the GVI (Gait Variability Index) showed a moderate reduction in the faller group ($M_{diff}=7.99,\;95\%\;CI\;[3.34,12.63]$), representing a medium effect size (d=0.59). When it comes to spatiotemporal variability, both SS ratio S.D. (d=0.58) and Toe In/Out Angle S.D. (d=0.44) demonstrated medium effects, suggesting increased instability during the single-limb loading phase. SS COP Distance S.D. (d=0.47) and Stride Width S.D. (d=0.39) also showed moderate differences, indicating less consistent lateral control in the fall-history group. Secondly, significant shifts in average gait patterns were observed, suggesting compensatory mechanisms or overall reduced mobility in the fall-prone group. The “faller” group walked notably slower, with a mean velocity difference of −14.60 [cm/s] (95% CI [−23.35, −5.86]) corresponding to a medium effect size (d=−0.57). This was accompanied by a reduction in Step Length Mean ($M_{diff}=-5.76$ [cm], d=−0.48). The DS COP Distance was higher in the fall-prone group ($M_{diff}=-2.74$ [cm], 95% CI [−5.12, −0.35]), with a small-to-medium effect size (d=−0.39), potentially reflecting an increased base of support during double stance to maintain balance. A comprehensive list of the significant gait parameters is summarized in Table 3.

### 3.4. Prediction of Faller from Walkway Gait Parameters

**Figure 2:**
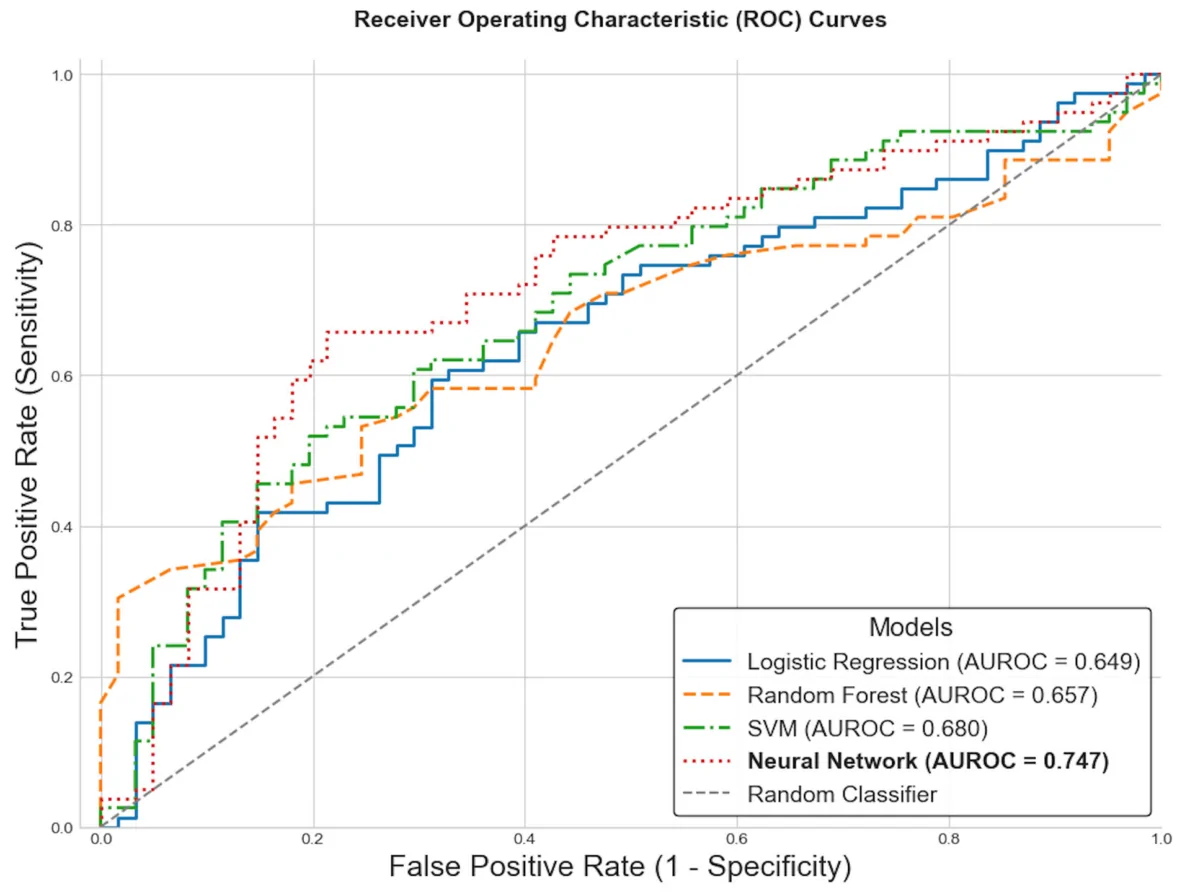
Comparison of receiver operating characteristic curves in faller prediction.

We preliminarily trained four machine learning models to classify individuals into “non-faller” or “faller” categories using gait parameters from the instrumented walkway. From the 10-fold cross-validation, the neural network model showed the highest overall performance across folds, with an area under the curve (AUC) of 0.747. The other models, including logistic regression, random forest, and SVM, demonstrated slightly lower but comparable performance. The fold-wise performance of these models evaluated across 10 folds is detailed in Table 4 and Figure 2.

## 4. Discussion

In this study, we investigated the relationship between gait parameters obtained during steady-state walking on an instrumented walkway and measures of balance impairment in a large, clinically diverse cohort of individuals with neurological disorders. Our findings demonstrate that instrumented gait analysis provides a rich source of data that is not only associated with balance impairment measured using a force plate but can also be used to predict it with acceptable accuracy. The results support our central hypothesis that spatiotemporal gait parameters measured during a simple walking task, a fundamental activity of daily life, can serve as a screening method for balance impairment, providing a convenient and efficient method of reducing the burden of performing balance-specific protocols. Our findings also generalize across a range of neurological disorders assessed. Crucially, in the context of advancing continuous real-world monitoring, these findings establish robust digital biomarkers that can guide the development of next-generation wearable sensing technologies.

A key finding was the profound difference in gait characteristics between the “non-faller” and “faller” groups, with 16 features showing statistical significance in Welch’s *t*-tests. The most impacted gait domain was gait variability, with significant differences in standard deviations of stride width, stride length, SS ratio, SS COP distance, CISP AP ratio, and Toe In/Out Angle. These findings align with extensive literature demonstrating that increased gait variability is a robust predictor of fall risk in older adults [50,51]. Specifically, stride-to-stride variability has been identified as an independent predictor of falls, potentially reflecting impaired neuromuscular control and reduced gait stability [52]. Step width variability, which showed a moderate effect size (d = 0.39), has been highlighted as having particularly strong discriminative power for identifying individuals at fall risk [50]. The GVI demonstrated a moderate effect size (d = 0.59), with fallers showing significantly higher values (133.74 ± 13.17) compared to non-fallers (125.76 ± 14.21). The GVI is a validated composite measure that captures variability across multiple spatiotemporal parameters and has been shown to differentiate between high-functioning older adults and those with mobility deficits. Higher GVI scores (indicating greater variability) are associated with impaired mobility function and balance performance [53].

In addition to the variability measures, spatiotemporal gait parameters also distinguished the groups. Fallers exhibited reduced velocity (72.27 vs. 86.87 [cm/s], d=−0.57) and shorter step length (44.56 vs. 50.33 [cm], d=−0.44), consistent with evidence that slower gait speed is one of the most reliable predictors of fall risk [6]. Clinical practice guidelines recommend using gait speed thresholds of <0.8–1.0 m/s for fall risk stratification and referral for prevention interventions [6,54]. The observed reduction in velocity among fallers likely reflects a cautious gait pattern. Interestingly, fallers demonstrated wider stride width (18.02 vs. 16.08 [cm], d=0.39), which may represent a compensatory strategy to enhance mediolateral stability. However, increased stride width has been associated with fall risk and mortality in some studies, suggesting this adaptation may be insufficient to prevent falls [52,55]. The reduced DS COP distance in fallers (39.82 vs. 42.56 [cm], d=−0.39) may indicate altered weight-transfer patterns during the double support phase, potentially reflecting compromised balance control during gait transitions.

These findings collectively suggest that gait variability measures, particularly when combined with reduced gait speed and altered spatial parameters, provide a comprehensive profile of fall risk in this population. The moderate-to-large effect sizes observed across multiple variability parameters underscore the clinical significance of these differences and support the use of comprehensive gait assessment for fall risk stratification [56,57,58,59].

Our machine learning models further solidified this connection. The ability of a neural network model to classify individuals with an AUC of 0.747 using only gait data is a promising result for clinical application. Given the limited number of samples as a preliminary study, the result suggests that data from a brief walk could be used as an objective, automated screening tool to flag individuals who may require a more comprehensive balance and falls risk assessment. This moderate discrimination aligns with recent literature indicating a performance ceiling (AUCs of roughly 0.62–0.70) when models rely strictly on standardized, clinically interpretable spatiotemporal features within clinically applicable recording length [57,58,59]. Although higher classification performance can be achieved using opaque signal transformations, these technical approaches sacrifice the clinical interpretability essential for routine care. To improve predictive performance without losing this interpretability, future work will focus on expanding the dataset to include a larger proportion of recurrent or frequent fallers, providing more robust class separation for training. Furthermore, a larger, balanced dataset will enable the exploration of more advanced neural network architectures beyond the traditional multilayer perceptron used here.

To ensure that our findings were not disproportionately driven by the large number of NPH patients in our cohort, we conducted a supplementary subgroup analysis comparing the NPH-only and non-NPH groups (see Supplementary Tables S1 and S2). This analysis confirmed that SS ratio variability remained significantly associated with faller status across both subgroups, indicating a generalized mechanism of balance impairment. Conversely, macroscopic spatiotemporal metrics like velocity and cadence were highly significant discriminators primarily within the NPH group, reflecting its classical hypokinetic gait phenotype. Within the non-NPH group, only three specific variables achieved statistical significance: SS ratio variability, DOP angle variability, and Toe In/Out Angle variability. Furthermore, our neural network classification performance remained highly robust when evaluated on these isolated subgroups, achieving an AUROC of 0.775 for the NPH-only dataset and 0.799 for the non-NPH dataset (compared to 0.747 for the overall dataset). This confirms that the model successfully utilizes generalized balance impairment signals to classify fallers, independent of the underlying diagnosis. It is important to note that the non-NPH group had a relatively small sample size and comprised multiple distinct conditions, i.e., CA, MS, and Parkinson’s disease. Therefore, the reduced statistical significance of some individual features in this specific subgroup may be partially attributable to limited statistical power and high clinical variance.

While gait parameters were strongly associated with the labels based on their falling history, their ability to directly predict objective force plate measures (COG mean velocity and COP Sway Area) was modest, with R^2^ values of 0.37 and 0.24, respectively. This does not diminish the value of gait analysis but rather highlights a crucial concept: static and dynamic balance are related but distinct constructs. Static balance primarily requires maintaining the body’s COM within a relatively fixed base of support. Conversely, dynamic balance during gait requires continuous, controlled shifting of the COM outside the base of support while in motion, necessitating active corrective stepping mechanisms. This fundamental difference in control mechanisms likely explains the modest predictive overlap between the two modalities. Eventually, these findings emphasize the need for dynamic monitoring solutions during gait analysis, which provide unique information about balance control during locomotion, which is the context in which most falls occur.

**Figure 3:**
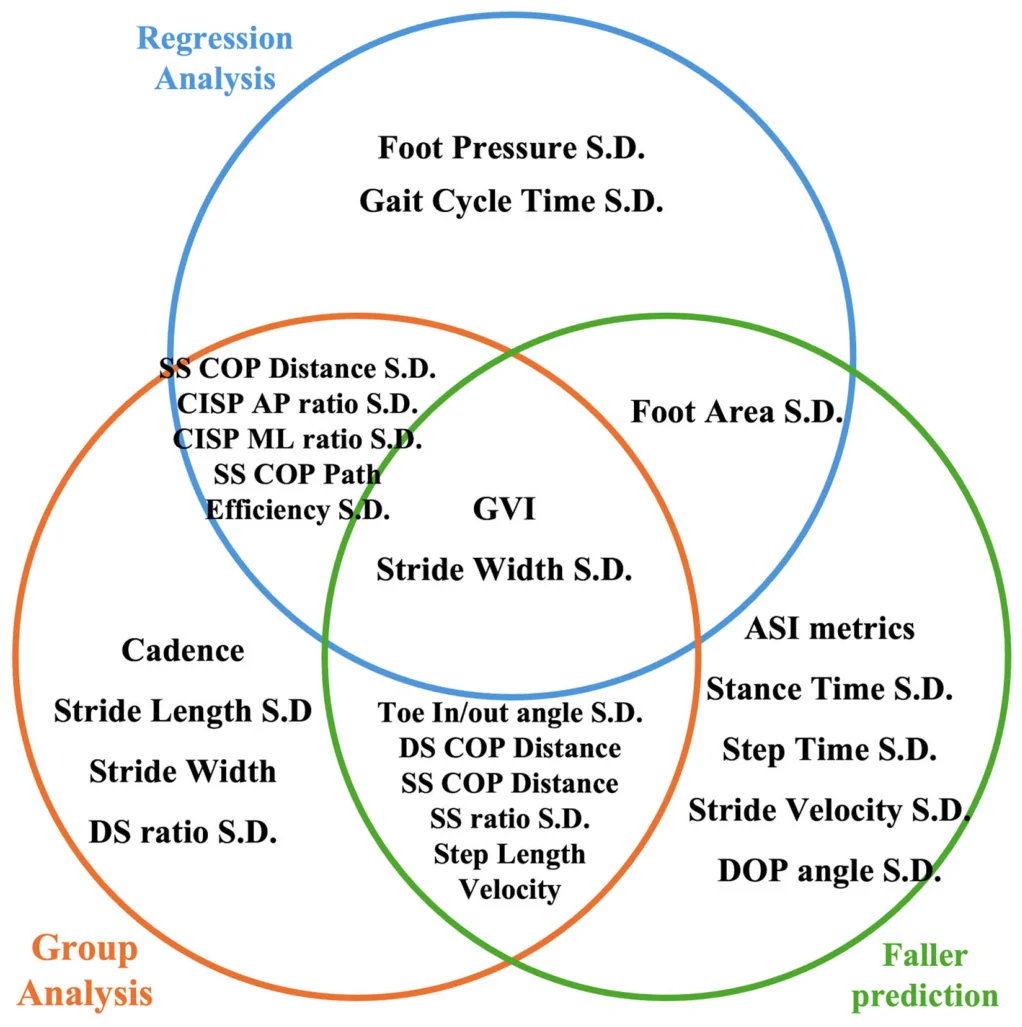
Venn diagram about convergence of predictive gait features across multi-perspective gait analysis.

Another important finding of this study is the convergence of key gait domains identified across all three distinct analytical approaches: the direct regression of force plate metrics, the group analysis between clinical balance impairment levels, and the machine learning classification models (Figure 3). This finding supports the idea that specific, quantifiable aspects of walking serve as powerful indicators of underlying balance control. Three primary themes consistently emerged as significant, directly informing the targets for future wearable engineering.

First, gait variability as measured by the standard deviation (S.D.) of various gait parameters was a dominant predictor across all tasks, though different analytical approaches highlighted complementary aspects. Stride Width S.D. and the Gait Variability Index (GVI) emerged as universal predictors, demonstrating significant discriminative and predictive power across all three analytical frameworks. Previous studies also reported that increased gait variability has been consistently linked to balance deficits and fall risk in older adults and neurological populations [60,61,62]. Higher step-time variability and movement trajectory amplitude in the medial–lateral and vertical directions are significantly correlated with impaired balance and increased fall risk in idiopathic normal pressure hydrocephalus [63]. Similarly, increased variability in step length during gait initiation is a strong predictor of postural problems and fall risk in elderly fallers [61]. In older adults, stance time and step width variability are associated with unique impairments in the central nervous system and sensory function, respectively, both of which contribute to balance impairment [60]. This suggests that individuals with poorer balance exhibit increased inconsistency and require more step-to-step corrective adjustments, reflecting a less natural and more unstable gait pattern.

Second, metrics of dynamic postural control during gait, particularly those involving the center of pressure (COP), were critically important. The group analysis highlighted generalized spatial variability and path metrics, showing statistically significant differences in features such as SS COP distance S.D., SS COP path efficiency S.D. and a reduction in overall DS COP distance and SS COP distance. However, the machine learning evaluation revealed a distinct, critical layer of impairment that the group comparisons missed: inter-limb asymmetry. Asymmetry indices (ASI) were the primary drivers of faller classification in the neural network. Stance COP distance mean—ASI was the single most important feature overall, closely followed by asymmetry in SS and DS COP distances. This indicates that while traditional statistics detect overall postural shifts, the machine learning model identifies the presence of compensatory, asymmetrical loading between limbs as a premier predictor of falls. Reduced dynamic stability, as measured by these metrics, is also known to be associated with increased gait variability and impaired balance in older adults and those with neurological conditions [63,64]. Patients with stroke, Parkinson’s disease, and traumatic brain injury demonstrate higher variability in gait stability indices and greater upper body instability, reflecting compromised dynamic postural control [3].

Finally, parameters related to foot placement and base of support, such as Stride Width and Toe In/Out Angle, were consistently identified as being related to balance function. The importance of the variability in these features, rather than just their mean values, underscores that balance impairment is characterized by an inability to maintain a consistent and effective foot placement strategy. Impaired foot placement control, as measured by deviation and adherence metrics, is consistently observed in individuals with balance impairments [65]. Mechanisms regulating foot placement and corrective ankle torque are essential for medial–lateral stability, and increased variability in these parameters is correlated with poor balance and higher fall risk [66].

In summary, increased gait variability, compromised dynamic postural control, and impaired foot placement parameters are all strongly associated with balance impairment and fall risk. The consistent identification of these gait signatures across multiple analyses strongly supports the use of these metrics as a tool for screening balance impairment. Future studies can use these identified metrics as digital biomarker targets for measurement using different sensor technologies and downstream development of personalized, data-driven adaptive rehabilitation strategies.

This study has several strengths, including a heterogeneous clinical population which replicates a realistic clinical neurology practice, a comprehensive set of gait parameters, and the use of both objective and expert-validated measures of balance. Our findings are generalizable across several neurological disorders assessed here, increasing the impact. However, some limitations must be acknowledged. First, the cross-sectional design prevents us from concluding causality or the ability of these gait features to predict future balance decline or falls. Future work should include longitudinal assessment of incident falls. Therefore, a critical next step is validating these identified digital biomarkers on an independent, external longitudinal dataset. Specifically, a prospective study tracking annual fall rates is necessary to establish their true clinical relevance for future incident fall prediction. Second, our reliance on retrospective self-reported questionnaires to ascertain fall history introduces the potential for recall bias, although prior studies evaluating a 12-month recall interval showed a high specificity of 91–95% and moderate sensitivity of 80–89% for falls [67]. The specific phrasing of the questions aimed to isolate unprovoked falls. To overcome this limitation, future research should incorporate objective, continuous fall tracking using wearable devices, such as smartwatches, during daily life. Third, our cohort focused on neurological disorders, and the findings may not be directly generalizable to a healthier aging population. Finally, the walking task was a simple, steady-state walk. However, walking in real life may pose added challenges such as obstacles and the need for multitasking. Our goal, however, was to identify the gait parameters that best capture balance. Therefore, future studies will be needed to validate the identified metrics in complex daily environments using validated wearable systems.

## 5. Conclusions

In conclusion, this study provides preliminary evidence that instrumented analysis of a simple walking task offers a practical and clinically accessible method for evaluating balance impairment. We found that gait variability is the key difference between fallers and non-fallers in this cohort of neurological conditions. Also, our preliminary classification model based on gait parameters, reflecting dynamic postural control, variability, and asymmetry, demonstrated the potential to detect balance deficits with moderate prediction accuracy, with an AUROC of 0.747. This study provides foundational knowledge that specific gait metrics can offer information on balance impairment. Future research is required to build upon this foundation and address current methodological challenges through larger, longitudinal studies in different disease groups to validate these gait biomarkers as predictors of future outcomes. Ultimately, by defining precisely which metrics to measure and their implications for balance and fall risk, these findings provide the basis for adapting other wearable sensing technologies into clinical practice, advancing the future of objective monitoring and personalized rehabilitation.

## Figures and Tables

**Table 1:** List of the selected gait parameters from PKMAS.

Gait Domain	Gait Parameters
Asymmetry	ASI of Stride Width, Stride Velocity, Gait Cycle Time, SS Ratio, DS Ratio, SS COP Distance, DS COP Distance, Stance COP Distance, Foot Area, Foot Pressure
Variability	SD of Stride Width, Stride Velocity, Stride Length, Step Time, SS Ratio, DS Ratio, Stance Time, SS COP Distance, SS COP Efficiency, Stance COP Efficiency, CISP AP Ratio, CISP ML Ratio, DOP Angle, Gait Cycle Time, Foot Area, Foot Angle, Foot Pressure, Toe In/Out Angle, GVI
Balance	Stride Width, Step Length, CISP AP Ratio, CISP ML Ratio, SS COP Efficiency, DS COP Distance, DS COP Efficiency, Stance COP Distance, Stance COP Efficiency, DOP Angle
Foot Contact	Foot Area, Foot Angle, Foot Pressure, Toe In/Out Angle
Rhythm	Cadence, SS Ratio, DS Ratio
Pace	Velocity, Step Length

**Table 2:** Demographic information.

	Entire Cohort (n: 140)	Group A: Non-Faller (n: 61)	Group B: Faller (n: 79)	Statistics	*p*-Value
Age [years]	66.98 ± 15.41 (28.0–88.0)	66.38 ± 15.80 (36.00–87.00)	67.44 ± 15.18 (28.00–88.00)	t = −0.403	0.69
Weight [kg]	85.20 ± 19.76 (46.70–136.90)	87.67 ± 20.26 (47.90–136.90)	83.30 ± 19.27 (46.70–122.70)	t = 1.291	0.20
Height [m]	1.72 ± 0.10 (1.49–1.97)	1.73 ± 0.11 (1.50–1.92)	1.71 ± 0.10 (1.49–1.97)	t = 0.982	0.33
Body mass index	28.67 ± 5.65 (17.59–46.18)	29.13 ± 5.37 (20.09–41.26)	28.32 ± 5.87 (17.59–46.18)	t = 0.844	0.40
Sex				χ^2^ = 2.513	0.11
Female	48 (34.3%)	16 (26.2%)	32 (40.5%)		
Male	92 (65.7%)	45 (73.8%)	47 (59.5%)		
Handedness				χ^2^ = 1.243	0.26
Right	76 (54.3%)	36 (59.0%)	40 (50.6%)		
Left	8 (5.7%)	6 (9.8%)	2 (2.5%)		
Unknown	46 (40.0%)	19 (31.1%)	37 (46.8%)		
Diagnosis				χ^2^ = 3.872	0.28
NPH	85 (60.7%)	39 (63.9%)	46 (58.2%)		
CA	22 (15.7%)	6 (9.8%)	16 (20.3%)		
MS	22 (15.7%)	12 (19.7%)	10 (12.7%)		
Parkinson’s Disease	11 (7.9%)	4 (6.6%)	7 (8.9%)		
Self-reported questionnaires					
Gait aid usage during day life				χ^2^ = 13.277	**0.01**
Do not require gait aid	71 (50.7%)	41 (67.2%)	30 (38.0%)		
Cane or walking stick	24 (17.1%)	9 (14.8%)	15 (19.0%)		
Walker	19 (13.6%)	5 (8.2%)	14 (17.7%)		
The help of a person	12 (8.6%)	2 (3.3%)	10 (12.7%)		
Wheelchair	14 (10.0%)	4 (6.6%)	10 (12.7%)		
Confidence about walking and balance abilities				χ^2^ = 37.323	**<0.001**
Not confident (0–29%)	16 (11.4%)	4 (6.6%)	12 (15.2%)		
Somewhat confident (30–59%)	49 (35.0%)	8 (13.1%)	41 (51.9%)		
Very confident (60–89%)	46 (32.9%)	25 (41.0%)	21 (26.6%)		
Highly confident (90–100%)	29 (20.7%)	24 (39.3%)	5 (6.3%)		

Continuous variables are presented as mean ± SD (range) and were compared between groups using Welch’s *t*-test. Categorical variables are presented as n (%) within each group and were compared using the chi-square test. For handedness, the test was performed on Left versus Right only. Bold denotes statistical significance. A *p*-value < 0.05 was considered statistically significant.

**Table 3:** Gait parameters with statistically significant differences between non-faller and faller groups in entire cohort.

Gait Parameter	Group A: Non-Faller (n: 61)	Group B: Faller (n: 79)	Mean Difference [95% CI]	Effect Size (Cohen’s d) [95% CI]
	Mean (SD)	Mean (SD)		
CISP AP ratio S.D.	3.50 ± 1.50	4.75 ± 2.07	1.25 [0.65, 1.85]	0.68 [0.33, 1.02]
GVI	125.76 ± 14.21	133.74 ± 13.17	7.99 [3.34, 12.63]	0.59 [0.24, 0.93]
SS ratio S.D.	2.07 ± 0.99	2.78 ± 1.40	0.71 [0.31, 1.11]	0.58 [0.23, 0.92]
Velocity	86.87 ± 26.27	72.27 ± 25.47	−14.60 [−23.35, −5.86]	−0.57 [−0.91, −0.23]
Step Length	50.33 ± 12.54	44.56 ± 11.73	−5.76 [−9.88, −1.65]	−0.48 [−0.82, −0.14]
SS COP Distance S.D.	1.40 ± 0.53	1.67 ± 0.64	0.28 [0.08, 0.47]	0.47 [0.13, 0.81]
Toe In/Out Angle S.D.	4.66 ± 1.46	5.58 ± 2.46	0.92 [0.26, 1.58]	0.44 [0.10, 0.78]
Stride Length S.D.	5.82 ± 1.84	6.68 ± 2.37	0.86 [0.16, 1.56]	0.40 [0.06, 0.74]
Cadence	101.26 ± 16.32	95.24 ± 14.46	−6.03 [−11.27, −0.78]	−0.39 [−0.73, −0.06]
Stride Width S.D.	2.18 ± 0.80	2.53 ± 0.97	0.35 [0.06, 0.65]	0.39 [0.05, 0.73]
DS COP Distance	42.56 ± 7.22	39.82 ± 6.85	−2.74 [−5.12, −0.35]	−0.39 [−0.73, −0.05]
Stride Width	16.08 ± 5.09	18.02 ± 4.90	1.94 [0.25, 3.63]	0.39 [0.05, 0.73]
SS COP Distance	9.43 ± 2.71	8.32 ± 3.08	−1.11 [−2.08, −0.14]	−0.38 [−0.72, −0.04]
CISP ML ratio S.D.	12.40 ± 11.59	16.52 ± 10.60	4.12 [0.35, 7.89]	0.37 [0.04, 0.71]
DS ratio S.D.	2.33 ± 1.57	2.92 ± 1.73	0.59 [0.04, 1.15]	0.36 [0.02, 0.69]
SS COP Path Efficiency S.D.	4.11 ± 6.83	6.53 ± 7.46	2.42 [0.02, 4.82]	0.34 [0.00, 0.67]
CISP AP ratio S.D.	3.50 ± 1.50	4.75 ± 2.07	1.25 [0.65, 1.85]	0.68 [0.33, 1.02]

**Table 4:** Comparison of fold-wise faller classification performance.

Model	Accuracy	Sensitivity	Specificity	F1-Sore	AUROC
Logistic Regression	0.61 ± 0.10	0.62 ± 0.15	0.61 ± 0.15	0.64 ± 0.13	0.65 ± 0.11
Randon Forest	0.63 ± 0.09	0.71 ± 0.15	0.52 ± 0.21	0.68 ± 0.10	0.66 ± 0.11
Support Vector Machine	0.64 ± 0.11	0.54 ± 0.17	0.75 ± 0.11	0.61 ± 0.16	0.68 ± 0.14
Neural Network	0.69 ± 0.07	0.77 ± 0.18	0.57 ± 0.20	0.72 ± 0.09	0.75 ± 0.07

## Data Availability

The data presented in this study are available on request from the corresponding author due to institutional privacy regulations and ethical restrictions protecting sensitive clinical information.

## Supplementary Materials

The following supporting information can be downloaded at: https://www.mdpi.com/article/10.3390/s26175644/s1, Table S1: Comparison of gait parameters between non-faller and faller groups within NPH subgroup. Table S2: Comparison of gait parameters between non-faller and faller groups within non-NPH subgroup.

## Abbreviations

The following abbreviations are used in this manuscript:

AP	Antero-Posterior
ASI	Asymmetry Index
AUC	Area Under the Curve
CA	Cerebellar Ataxia
CISP	Cyclogram Intersection Point
COG	Center of Gravity
COM	Center of Mass
COP	Center of Pressure
DOP	Direction of Progression
DS	Double Support
GVI	Gait Variability Index
IMU	Inertial Measurement Unit
LASSO	Least Absolute Shrinkage and Selection Operator
mCTSIB	Modified Clinical Test of Sensory Integration and Balance
ML	Medio-Lateral
MS	Multiple Sclerosis
NPH	Normal Pressure Hydrocephalus
S.D.	Standard Deviation
SS	Single Support
SVM	Support Vector Machine
