# Supplementary material for: Instrumented Walkway Gait Analysis Predicts Fallers in Neurological Disorders: Identifying Digital Biomarkers for Balance Monitoring
Source: Sensors (Basel). 2026 Sep 5;26(17):5644. doi: 10.3390/s26175644 (PMC13568298; doi:10.3390/s26175644)
Supplement: Supplementary file 1 [file sensors-26-05644-s001.zip › sensors-4485843-supplementary.pdf]

Supplementary Table S1. Comparison of gait parameters between non-faller and faller groups within NPH subgroup

Gait parameter	Group A: Non-faller (n: 39) Mean (SD)	Group B: Faller (n: 46) Mean (SD)	Mean Difference [95% CI]	Effect Size (Cohen's d) [95% CI]
**CISP AP ratio S.D.**	3.52 ± 1.10	4.84 ± 1.77	1.33 [0.70, 1.96]	**0.89* [0.44, 1.33]**
**GVI**	125.98 ± 10.41	135.06 ± 11.44	9.08 [4.37, 13.80]	**0.83* [0.38, 1.27]**
**SS ratio S.D.**	2.05 ± 0.81	2.61 ± 1.19	0.55 [0.12, 0.99]	0.54 [0.10, 0.97]
**Velocity**	83.97 ± 20.77	65.15 ± 22.71	-18.82 [-28.20, -9.43]	**-0.86* [-1.31, -0.42]**
**Step Length**	48.32 ± 10.79	41.11 ± 11.21	-7.21 [-11.96, -2.45]	**-0.65* [-1.09, -0.22]**
SS COP Distance S.D.	1.39 ± 0.49	1.59 ± 0.45	0.20 [-0.00, 0.41]	0.43 [0.00, 0.87]
Toe In/Out Angle S.D.	4.76 ± 1.34	5.52 ± 2.58	0.76 [-0.11, 1.63]	0.36 [-0.07, 0.79]
**Stride Length S.D.**	5.82 ± 1.59	6.64 ± 2.16	0.82 [0.01, 1.63]	0.43 [-0.00, 0.86]
**Cadence**	103.63 ± 12.01	93.17 ± 14.16	-10.47 [-16.11, -4.82]	**-0.79* [-1.23, -0.35]**
Stride Width S.D.	2.07 ± 0.48	2.36 ± 0.86	0.29 [-0.01, 0.59]	0.41 [-0.02, 0.84]
DS COP Distance	41.33 ± 7.05	38.41 ± 7.04	-2.93 [-5.98, 0.12]	-0.42 [-0.85, 0.02]
**Stride Width**	16.33 ± 4.16	18.54 ± 4.93	2.21 [0.25, 4.17]	**0.48* [0.05, 0.91]**
**SS COP Distance**	9.04 ± 2.34	7.37 ± 3.03	-1.67 [-2.83, -0.51]	**-0.61* [-1.05, -0.18]**
**CISP ML ratio S.D.**	11.44 ± 5.59	17.77 ± 10.14	6.33 [2.86, 9.80]	**0.76* [0.31, 1.20]**
**DS ratio S.D.**	2.16 ± 0.86	3.08 ± 1.85	0.92 [0.31, 1.53]	**0.62* [0.18, 1.06]**
**SS COP Path Efficiency S.D.**	3.34 ± 4.43	6.87 ± 7.51	3.53 [0.92, 6.15]	**0.56* [0.13, 1.00]**
**CISP AP ratio S.D.**	3.52 ± 1.10	4.84 ± 1.77	1.33 [0.70, 1.96]	**0.89* [0.44, 1.33]**
**SS ratio**	32.94 ± 3.18	30.33 ± 4.15	-2.61 [-4.19, -1.03]	**-0.70† [-1.14, -0.26]**
**Stance Time S.D.**	0.05 ± 0.02	0.11 ± 0.14	0.06 [0.02, 0.10]	**0.55† [0.11, 0.98]**
**Step Time S.D.**	0.04 ± 0.02	0.08 ± 0.10	0.04 [0.01, 0.07]	**0.52† [0.09, 0.96]**
**Foot Pressure S.D.**	27.23 ± 10.36	40.18 ± 34.61	12.95 [2.19, 23.71]	**0.49† [0.06, 0.92]**
**Stride Width - ASI**	0.92 ± 2.05	0.08 ± 1.69	-0.83 [-1.66, -0.01]	**-0.45† [-0.88, -0.02]**

Bold text indicates gait parameters showed statistical significance between non-faller and faller groups. * Gait parameters that also showed statistical significance differences in the entire cohort. † Gait parameters showed statistical significance only within NPH subgroup.

Supplementary Table S2. Comparison of gait parameters between non-faller and faller groups within non-NPH subgroup

Gait parameter	Group A: Non-faller (n: 22) Mean (SD)	Group B: Faller (n: 33) Mean (SD)	Mean Difference [95% CI]	Effect Size (Cohen's d) [95% CI]
CISP AP ratio S.D.	3.48 ± 2.05	4.62 ± 2.46	1.14 [-0.09, 2.37]	0.49 [-0.05, 1.04]
GVI	125.36 ± 19.52	131.90 ± 15.26	6.54 [-3.46, 16.54]	0.38 [-0.16, 0.93]
**SS ratio S.D.**	2.09 ± 1.27	3.01 ± 1.63	0.92 [0.14, 1.71]	**0.62* [0.07, 1.17]**
Velocity	92.03 ± 33.88	82.19 ± 26.10	-9.83 [-27.12, 7.46]	-0.33 [-0.88, 0.21]
Step Length	53.88 ± 14.75	49.37 ± 10.86	-4.51 [-11.95, 2.93]	-0.36 [-0.90, 0.18]
SS COP Distance S.D.	1.41 ± 0.61	1.78 ± 0.83	0.38 [-0.01, 0.77]	0.50 [-0.04, 1.05]
**Toe In/Out Angle S.D.**	4.47 ± 1.67	5.65 ± 2.33	1.18 [0.09, 2.26]	**0.56* [0.01, 1.11]**
Stride Length S.D.	5.80 ± 2.27	6.72 ± 2.66	0.92 [-0.42, 2.26]	0.37 [-0.18, 0.91]
Cadence	97.06 ± 21.70	98.12 ± 14.59	1.06 [-9.67, 11.79]	0.06 [-0.48, 0.60]
Stride Width S.D.	2.35 ± 1.17	2.76 ± 1.08	0.40 [-0.23, 1.03]	0.36 [-0.18, 0.90]
DS COP Distance	44.73 ± 7.16	41.80 ± 6.14	-2.93 [-6.70, 0.83]	-0.45 [-0.99, 0.10]
Stride Width	15.63 ± 6.51	17.29 ± 4.83	1.66 [-1.63, 4.95]	0.30 [-0.24, 0.84]
SS COP Distance	10.12 ± 3.21	9.63 ± 2.69	-0.48 [-2.16, 1.19]	-0.17 [-0.71, 0.37]
CISP ML ratio S.D.	14.11 ± 17.97	14.78 ± 11.12	0.67 [-8.07, 9.42]	0.05 [-0.49, 0.59]
DS ratio S.D.	2.62 ± 2.35	2.70 ± 1.54	0.08 [-1.08, 1.23]	0.04 [-0.50, 0.58]
SS COP Path Efficiency S.D.	5.49 ± 9.73	6.06 ± 7.48	0.57 [-4.39, 5.54]	0.07 [-0.47, 0.61]
CISP AP ratio S.D.	3.48 ± 2.05	4.62 ± 2.46	1.14 [-0.09, 2.37]	0.49 [-0.05, 1.04]
**DOP Angle S.D.**	103.76 ± 31.93	115.39 ± 5.27	11.63 [-2.62, 25.89]	**0.57† [0.02, 1.12]**

Bold text indicates gait parameters showed statistical significance between non-faller and faller groups. * Gait parameters that also showed statistical significance differences in the entire cohort. † Gait parameters showed statistical significance only within non-NPH subgroup.
